# E-cigarette aerosols induce unfolded protein response in normal human oral keratinocytes

**DOI:** 10.7150/jca.31319

**Published:** 2019-11-19

**Authors:** Eoon Hye Ji, Naseim Elzakra, Wei Chen, Li Cui, Eon S Lee, Bingbing Sun, Diana Messadi, Tian Xia, Yifang Zhu, Shen Hu

**Affiliations:** 1 School of Dentistry, University of California, Los Angeles, CA 90095, USA; 2 Fielding School of Public Health, University of California, Los Angeles, CA 90095, USA; 3 Division of Nanomedicine, School of Medicine, University of California, Los Angeles, CA 90095, USA; 4 State Key Laboratory of Fine Chemicals, School of Chemical Engineering, Dalian University of Technology, 2 Linggong Road, 116024, Dalian, China

**Keywords:** E-cigarette aerosols, protein response, human oral keratinocytes

## Abstract

**Objective**: Since the introduction in 2004, global usage of e-cigarettes (ECs) has risen exponentially. However, the risks of ECs on oral health are uncertain. The purpose of this study is to understand if EC aerosol exposure impacts the gene pathways of normal human oral keratinocytes (NHOKs), particularly the unfolded protein response (UPR) pathway.

**Materials and methods:** EC aerosols were generated reproducibly with a home-made puffing device and impinged into the culture medium for NHOKs. DNA microarrays were used to profile the gene expression changes in NHOKs treated with EC aerosols, and the Ingenuity Pathway Analysis (IPA) was used to reveal signaling pathways altered by the EC aerosols. Quantitative PCR was used to validate the expression changes of significantly altered genes.

**Results:** DNA microarray profiling followed by IPA revealed a number of signaling pathways, such as UPR, cell cycle regulation, TGF-β signaling, NRF2-mediated oxidative stress response, PI3K/AKT signaling, NF-κB signaling, and HGF signaling, activated by EC aerosols in NHOKs. The UPR pathway genes, C/EBP homologous protein (CHOP), activating transcription factor 4 (ATF4), X box binding protein 1 (XBP1), and inositol-requiring enzyme 1 alpha (IRE1α) were all significantly up-regulated in EC aerosol-treated NHOKs whereas immunoglobulin heavy-chain binding protein (BIP) and PRKR-like ER kinase (PERK) were slightly up-regulated. qPCR analysis results were found to be well correlated with those from the DNA microarray analysis. The most significantly changed genes in EC aerosol-treated NHOKs *versus* untreated NHOKs were CHOP, ATF4, XBP1, IRE1α and BIP. Meanwhile, Western blot analysis confirmed that CHOP, GRP78 (BIP), ATF4, IRE1α and XBP1s (spliced XBP1) were significantly up-regulated in NHOKs treated with EC aerosols.

**Conclusion**: Our results indicate that EC aerosols up-regulate the UPR pathway genes in NHOKs, and the induction of UPR response is mediated by the PERK - EIF2α - ATF4 and IRE1α - XBP1 pathways.

## Introduction

Electronic cigarettes (ECs), also known as vapor cigarettes or vaporizer pens, are battery-powered devices that provide an inhalant containing nicotine and other additives. Most of the ECs are made with a heating element which generates aerosols from a E-liquid solution at a temperature of roughly 100 - 250°C 1. Recently, there has been a dramatic increase in EC usage, mainly because ECs deliver nicotine with flavorings to users in vapor instead of smoke, which is much less prohibited in public areas. Many have switched from conventional cigarettes believing ECs had little or no risk 2. Based on a survey study, about 2.4 million middle and high school students in the US are exposed to ECs. Strikingly, among the US high school students, the use of ECs has been increased from 1.5% to 13.4% based on a recent report 3. Despite the consideration of ECs as a potential substitute smoking device for conventional smoking, many studies have investigated the behavior of EC users or pathological symptoms. However, these studies were mainly based on a short-term EC use 2, 4. Generating data from long-term EC use is necessary to establish guidelines and regulatory decisions on EC production and usage.

Few studies have been carried out to investigate the effect of ECs on the oral cavity. Most of the current studies, in regards to EC research at the molecular level, are focused on relationships between EC and lung/airway epithelia cells or tissues. Lerner *et al.*, stated that when lung cells and tissues were exposed to EC aerosols, oxidative and inflammatory responses occurred 5. Sussan *et al.* demonstrated that, when mice were infected with *Streptococcus pneumonia* and exposed to EC aerosols, their pulmonary bacterial clearance was impaired significantly compared to air-exposed mice 6. EC aerosols allow lung epithelia cells to be very susceptible to viral infections and cause weakened immune system. A recent study showed that exposure to EC aerosol mixtures with flavorings increased oxidative/carbonyl stresses and inflammatory cytokine release in human periodontal ligament fibroblasts, human gingival epithelium progenitors, and 3D EpiGingival tissues 7. In our previous study, we characterized EC aerosols using a combination of advanced technologies. Our findings suggested that EC aerosols induce cytotoxicity to oral epithelial cells *in vitro*, which may be partially mediated by oxidative stress response 8. Oxidative stress response may lead to numerous changes in the cells including gene expression changes depending on the level of oxidative stress. To the best of our knowledge, there has not been systematic analysis of the gene expression changes in oral epithelial cells caused by EC aerosols.

In this study, we have investigated the effects of EC aerosols on gene expression changes in normal human oral keratinocytes (**NHOKs**) and found that UPR is significantly activated by EC aerosols. In response to a variety of pathological stimuli, such as aerosols from cigarettes and possibly EC, nutrient deprivation, oxidative stress, DNA damage, or energy perturbation or fluctuations 9, endoplasmic reticulum (**ER**) stress may occur in eukaryotic cells and result in unfolded or misfolded proteins. This leads to the activation of a cascade of intracellular signaling molecules of the UPR pathway. UPR plays important role in restoring homeostasis, degrading misfolded proteins, and triggering cellular signals to assist protein folding. However, it may also lead to apoptosis if protein misfolding is not fixed 10. Through DNA microarray and qPCR/Western blot analyses, we have demonstrated that EC aerosols activate the molecular determinants of the UPR signaling pathway in NHOKs.

## Materials and Methods

### Cell culture

NHOKs were maintained at 37 °C in an atmosphere of 95% air and 5% CO_2_. The cells were passaged at 60-70%confluency level and allowed to proliferate in the EpiLife medium supplemented with the human keratinocyte growth supplement (Invitrogen, Carlsbad, CA), as described previously(Ji et al., 2016). When the NHOKs reached 60-70% confluence, the cells were washed once with PBS and subsequently were incubated with the EC aerosol-impinged EpiLife culture media in the CO_2_ incubator. Generation of EC aerosols and EC aerosol-impinged cultured media are described below in details.

### Generation of EC aerosols

**Figure 1:**
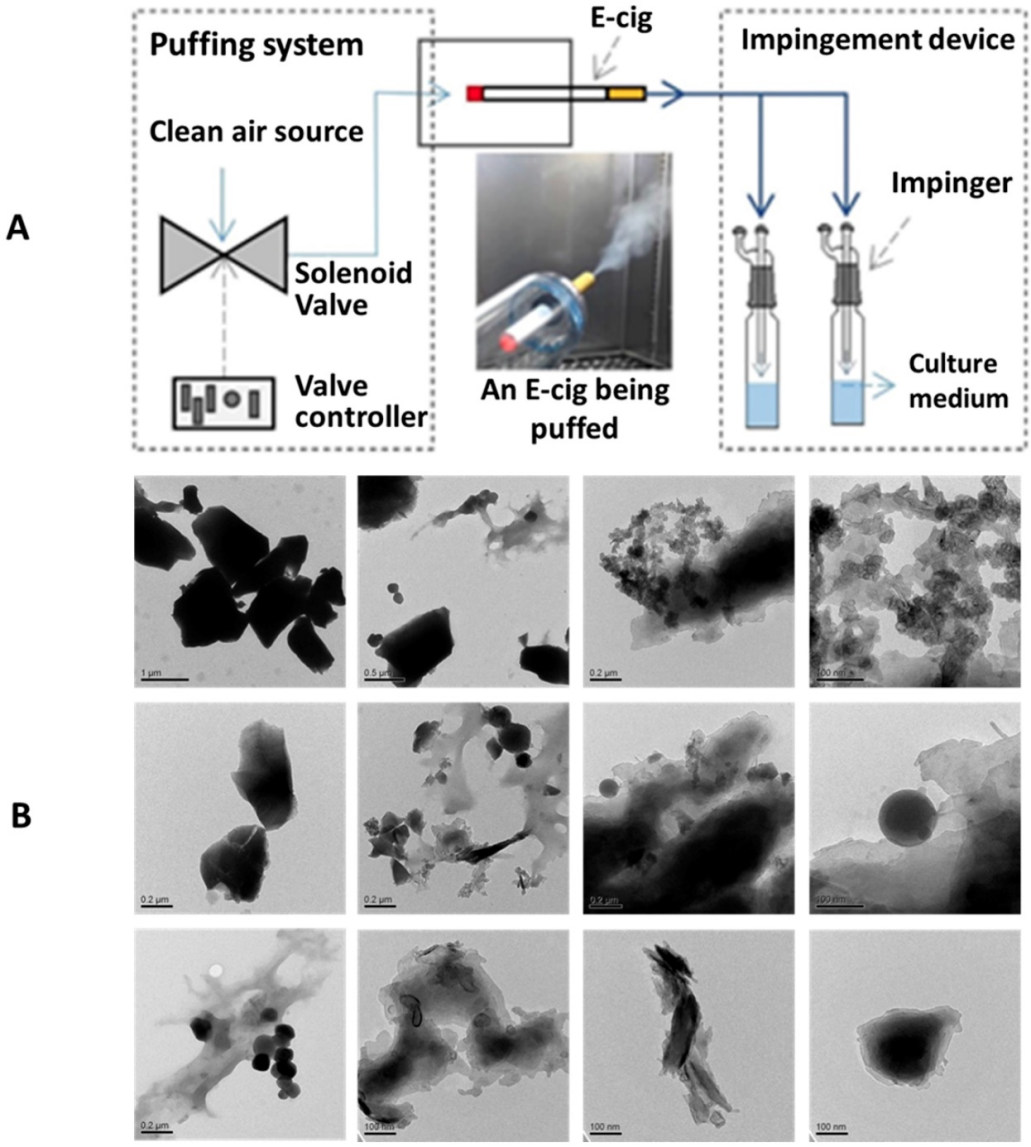
** (A)** A schematic diagram of the apparatus to generate EC aerosols and impinge the cell culture medium. **(B)** TEM images of EC aerosol microparticles/nanoparticles.

EC aerosols were generated from an e-liquid mixture using an EC emission apparatus (**Figure 1**). The e-liquid mixture was prepared from individual chemical compounds of propylene glycol (PG, C_3_H_8_O_2_, ≥ 99.5%), vegetable glycerin (VG, C_3_H_8_O_3_, ≥ 99.5%), and nicotine (Nic, C_10_H_14_N_2_, ≥ 99%). The e-liquid mixture used in this study had a 29.3% PG and 68.3% VG with 2.4 mg Nic/l. With the e-liquid mixture, e-cig aerosol emissions were generated by using a third generation EC device, *so-called* “Mods” (*i.e.,* Vapor-fi model Volt Hybrid Tank used in this study). This type of EC device is selected because of its high popularity among the EC devices used.EC aerosols were generated with a thermal heating coil (0.5 Ω) in the EC device at a constant 7.5 W electrical power. Particle-free (i.e., HEPA-filtered) air was supplied to the EC device at 1 l/min airflow rate. The generated EC aerosols were collected in a series of three glass impingers. The impinged EC aerosol concentration per 1ml of medium used was: 14.89 mg EC aerosol per ml medium. High throughput dynamic light scattering (HT-DLS, Dynapro™ Plate Reader, Wyatt Technology) was performed to determine the particle size and size distribution of the EC aerosols in aqueous solution. Transmission electron microscopy (TEM, JEOL 1200 EX, accelerating voltage 80 kV) was used to determine the morphology and primary size of EC aerosol nanoparticles.

### Treatment of NHOKs with EC aerosols

EC aerosols were prepared as described above and immediately impinged into the NHOK culture media during 15 minutes. The particle suspensions were sonicated for 5 min using a water bath sonicator to obtain well-dispersed particle suspensions. Afterwards, the impinged culture medium was immediately used to treat NHOKs. After the NHOKs (on petri dish, ~80% confluence) were washed once with PBS, the impinged culture medium was added to the petri dish and incubated with the cells for 4 hours (5% CO_2_, 37 °C) prior to harvesting for DNA microarray and qPCR analyses.

### DNA microarray analysis

RNA was extracted using the Qiagen RNAeasy Micro Kit, following the manufacturer's instruction. RNA purity/concentration was determined using a Nanodrop 8000 (Thermo Fisher, Waltham, MA), and RNA integrity was analyzed using an Agilent 2100 Bioanalyzer (Agilent Technologies, Palo Alto, CA). Microarray targets were generated using the FL-Ovation cDNA Biotin Module V2 (NuGen Technologies, San Carlos, CA) and then hybridized to the Affymetrix Gene Chip U133Plus 2.0 Array (Affymetrix, Santa Clara, CA), which contains > 54,000 probe sets representing > 47,000 transcripts and variants, according to the manufacturers' instructions. The arrays were washed and stained with streptavidin phycoerythrin in Affymetrix GeneChip protocol, and then scanned using an Affymetrix GeneChip Scanner 3000. Microarray analysis was performed in duplicates and the AGCC software (Affymetrix) was utilized for acquisition of array images and initial quantification.

### Quantitative real-time PCR

Total RNA was extracted from cultured cells using the RNeasy Mini Kit (Qiagen, Valencia, CA). cDNA conversion was carried out using Superscript II reverse transcriptase (Invitrogen), and 5μg of RNA per sample was converted. mRNA and cDNA concentration was measured using a Nanodrop spectrometer (Thermo Fisher). cDNA concentration of test and control samples was diluted to equal concentration (100ng/μl). For qPCR reaction, 1μl of diluted cDNA solution was mixed with 0.4μl primer, 10μl DEPC-treated RNAase/DNA free water and 8.6μl SYBR Green I MasterMix (Roche, Indianapolis, IN) in a 96-well PCR plate and the reaction was performed on a CFX96 qPCR system (Bio-Rad, Hercules, CA). Actin was used as a housekeeping Gene for normalization. Three biological replicates were analyzed with qPCR and the data was analyzed using the 2^-ΔΔCt^ method. **Table 1** lists the sequences for the primers used in this study for qPCR analysis.

### Western blot analysis

Protein samples were separated with a 4-12% Bis-Tris NuPAGE gel (Invitrogen) and transferred onto nitrocellulose membrane by the Trans-blot SD semi-dry transfer cell (Bio-Rad, Brea, CA, USA). The membranes were blocked in TBST buffer containing 5% nonfat milk (Santa Cruz Biotech), and incubated with antibodies against human protein (anti-GRP78, anti-CHOP, anti-IRE1a, anti-XBP1, anti-ATF4 and anti-ATF6a) at a dilution of 1:500 (Santa Cruz Biotech) overnight, followed by HRP linked anti-mouse or anti-rabbit IgG (1:5000; GE Healthcare). The detection was performed with the ECL-Plus Western blotting reagent kit (GE Healthcare). Western blot analysis was performed in triplicates.

### Statistical analysis

The data were expressed as the mean ± standard deviation, and analyzed by the independent samples t-test using the MedCalc (MedCalc Software, Ostend, Belgium). P values < 0.05 were considered as statistically significant. Differentially expressed genes were selected at ≥2-fold difference and P < 0.05. Functional pathway analysis of the genes at significantly altered levels (derived from DNA microarray analysis) was performed with the Ingenuity Pathway Analysis (**IPA**, Qiagen). The Ingenuity Pathways Knowledge Base (IPKB) provided all the published known functions and interactions. Fischer's exact test was used to calculate a P-value to determine the significance of each canonical pathway.

## Results

### Generation of EC aerosols

We have utilized a home-made EC puffing apparatus to generate the EC aerosols for *in vitro* experiments. As shown in **Figure 1A**, a puffing controller comprised of clean compressed air source, flow valve, and valve timer was used to push air through the tip and out of the mouth piece of an EC, to simulate reproducible EC aerosol delivery process. The generated EC aerosols were impinged into cell culture medium and, in 30 minutes, the impinged culture media were used to treat the NHOKs for 4 hours. **Figure 1B** shows the transmission electron microscope (**TEM**) image of EC aerosols, revealing the presence of flake-like and spherical micro-and nano-particles in EC aerosols. The size of EC aerosol nanoparticles in liquid phase varied significantly from ~ 100 nm to ~ 1 µm.

### DNA microarray analysis of EC aerosol-treated NHOKs

**Figure 2:**
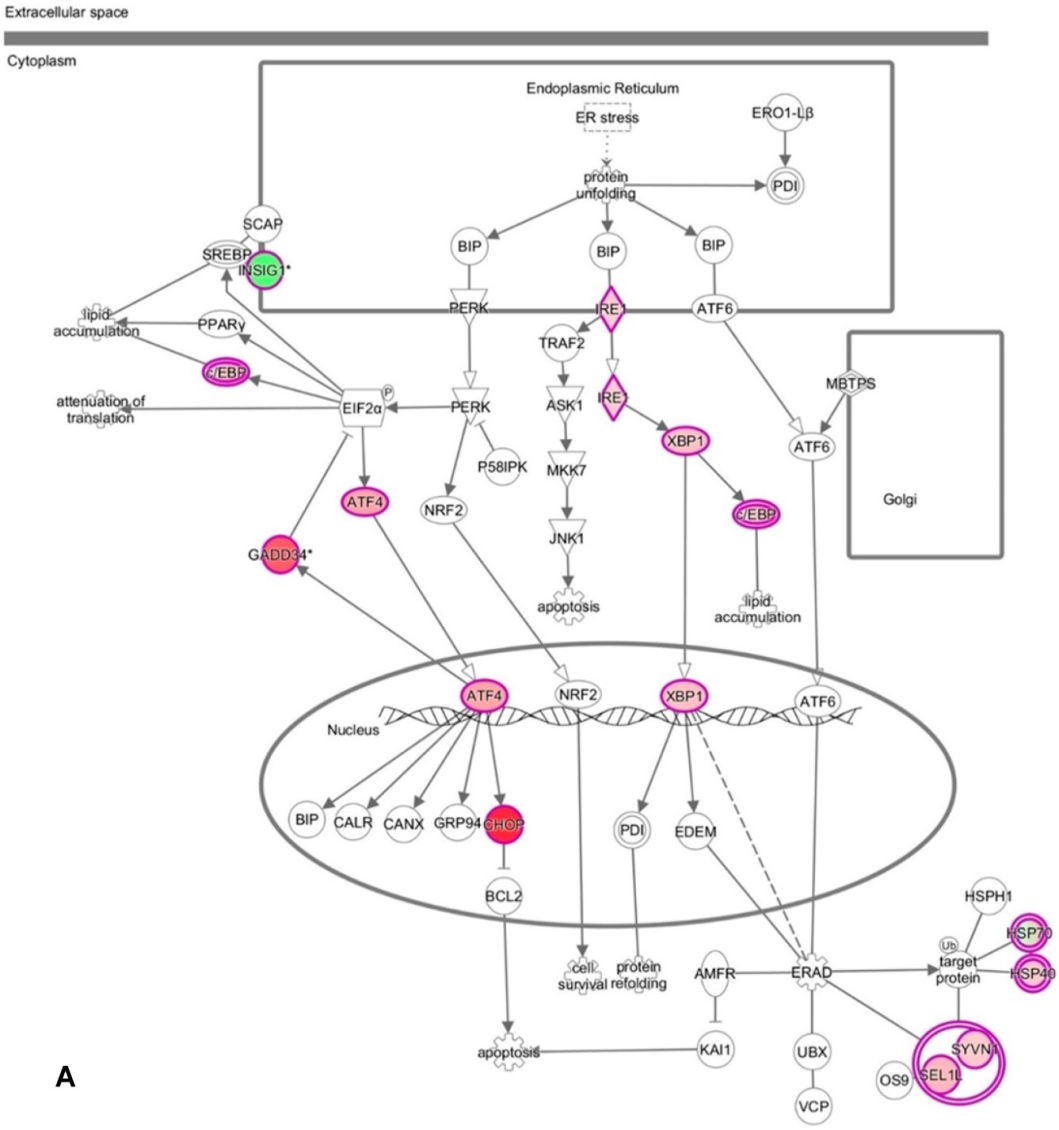

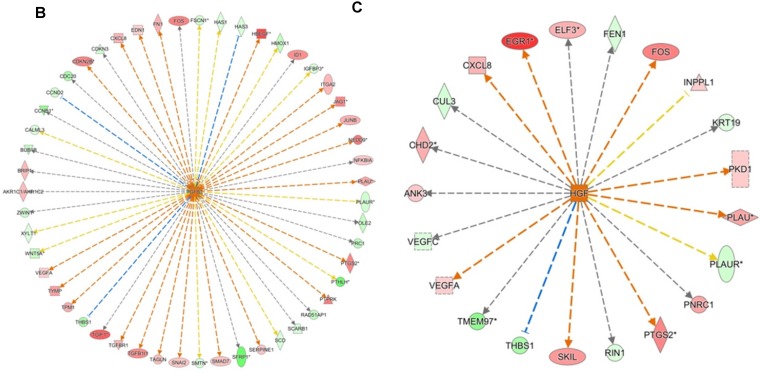
DNA microarray analysis followed by functional pathway analysis revealed unfolded protein response (UPR) pathways activated in normal human oral keratinocytes (NHOKs) by EC aerosols. (A) The canonical UPR pathway was generated with the Ingenuity Pathways Analysis (IPA) software. A number of genes of the UPR pathways such as XBP1 (X-box binding protein 1), IRE1α (inositol-requiring enzyme 1, a.k.a., endoplasmic reticulum to nucleus signaling 1), ATF4 (activating transcription factor 4), CHOP (a.k.a., DDIT3, DNA-damage-inducible transcript 3) and C/EBP were induced by EC aerosols. (B & C) TGF-β and HGF signals activated in EC aerosol-treated NHOKs.

We have profiled the gene expression changes in NHOKs treated with EC aerosols using the Human Genome U133 Plus 2.0 microarrays (Affymetrix). In total, 2350 genes were found to be significantly changed at 2-fold or more in EC aerosol-treated NHOKs when compared to untreated cells. Functional pathway analysis of the genes at significantly altered levels with the Ingenuity Pathway Analysis (**IPA**) indicated that many signaling pathways were activated in NHOKs due to the exposure to EC aerosols (**Table 2**). Among them, the UPR pathway was found to be ranked on the top. As shown in the UPR gene network generated by the IPA (**Figure 2A**), important molecular determinants of the UPR pathway such as activating transcription factor 4 (ATF4), C/EBP homologous protein (CHOP, *a.k.a*., DNA-damage-inducible transcript 3), X box binding protein 1 (XBP1), and inositol-requiring enzyme 1 alpha (IRE1α, *a.k.a*., endoplasmic reticulum to nucleus signaling 1) were all activated in EC aerosol-treated NHOKs. Other types of signaling pathways such as TGF-β and HGF were also activated in EC aerosol treated NHOKs (**Figure 2B and 2C**). TGF beta pathway is involved in many cellular processes such as cell growth, cell differentiation, and apoptosis whereas HGF pathway is related to cell proliferation, differentiation, and motility. A list of UPR pathway genes at differential expression levels between EC aerosol-treated and untreated NHOKs are shown in **Table 3**. CHOP (fold change, 9.60), ATF4 (fold change, 3.65), and XBP1 (fold change, 2.52) were most significantly changed genes. However, immunoglobulin heavy-chain binding protein (BIP, *a.k.a*., glucose-regulated protein 78, fold change = 1.68) and PRKR-like ER kinase (PERK, fold change = 1.35) were modestly up-regulated whereas activating transcription factor 6α (ATP6α, fold change = 1.01) was almost unchanged.

### qPCR analysis of UPR pathway genes in EC aerosol-treated NHOKs

**Figure 3:**
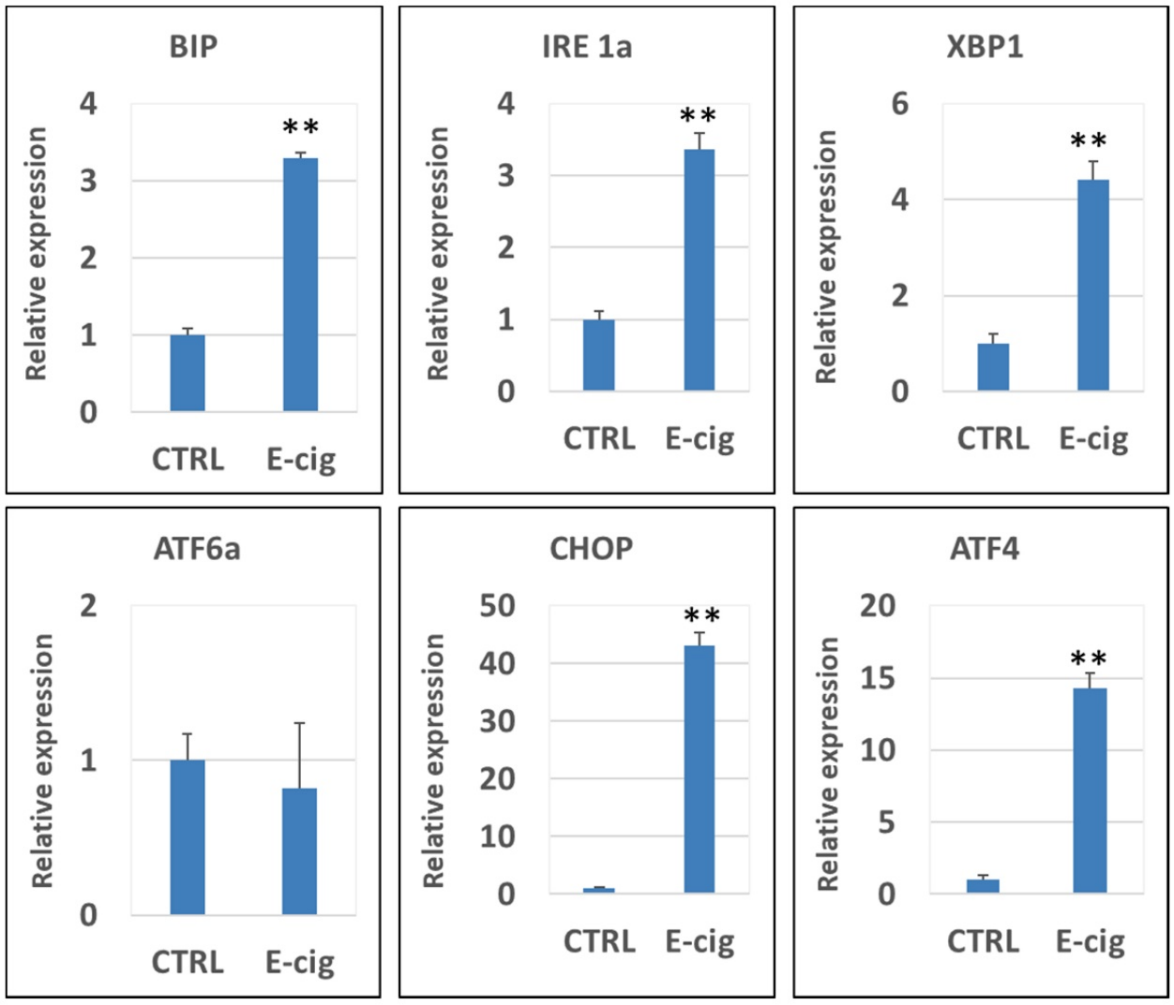
qPCR validation of gene expression of the UPR pathway in NHOKs treated with E-cig aerosols (n=3). CHOP, ATF4, XBP1 and IRE1α and BIP were significantly up-regulated in NHOKs after the EC aerosol exposure (**, p < 0.01; fold change > 3). However, ATF6 was not significantly altered by EC aerosols. The primers used for qPCR analysis were listed in Table 1.

**Figure 3** shows the qPCR analysis results of the gene expression levels of CHOP, XBP1, ATF4, IRE1α, BIP, PERK and ATF6α in NHOKs treated with EC aerosols in relative to untreated NHOKs. The most significantly changed genes were CHOP (fold change, 43.1), ATF4 (fold change, 14.3) and XBP1 (fold change, 4.42) according to the qPCR analysis. Meanwhile, PERK was slightly up-regulated (fold change, 1.56) whereas ATF6α was not significantly changed. However, BIP was found to be significantly over-expressed (fold change, 3.29) in EC aerosol-treated NHOKs compared to untreated NHOKs. As shown in Table 3 and Figure 3, DNA microarray and qPCR analyses showed consistency on the expression changes of all genes except BIP. DNA microarray analysis indicated that BIP was modestly up-regulated in EC aerosol-treated cells but the gene was found to be significantly up-regulated by qPCR analysis. Nevertheless, these results confirmed that EC aerosols activate the expression of UPR pathway genes in NHOKs.

### Western blot analysis of UPR pathway gene products in EC aerosol-treated NHOKs

**Figure 4:**
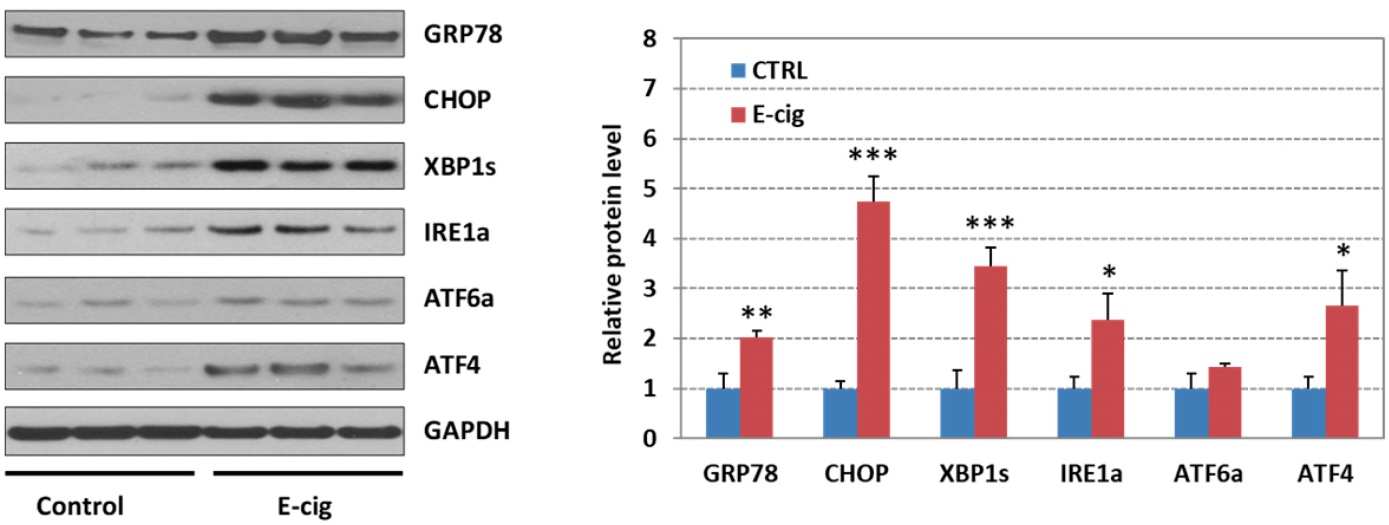
Western blot analysis of GRP78 (BIP), CHOP, IRE1α, XBP1s (spliced XBP1), ATF4 and ATF6 in NHOKs treated with EC aerosols (n=3, *, p < 0.05; **, p < 0.01). The results confirmed that GRP78, CHOP, IRE1α, XBP1s and ATF4 were significantly up-regulated in NHOKs by EC aerosols.

**Figure 4** shows the Western blot analysis of GRP78 (BIP), CHOP, XBP1s (spliced XBP1), ATF4, IRE1α, and ATF6α in EC aerosol-treated *versus* untreated NHOKs. The results indicated that EC aerosol treatment caused significant changes in protein levels of GRP78 (fold change, 2.03), CHOP (fold change, 4.74), ATF4 (fold change, 2.67), XBP1s (fold change, 3.44), and IRE1α (fold change, 2.37). Meanwhile, ATF6 was not significantly up-regulated (fold change, 1.42). These results further confirmed that EC aerosols activate the expression of UPR pathway genes in NHOKs.

## Discussion

Upon ER stress, unfolded and misfolded proteins bind and sequester BIP protein (*a.k.a.*, GRP78), thereby activating the UPR. The UPR comprises three parallel signaling branches: PERK (EIF2AK) - eukaryotic translation initiation factor 2α (EIF2α) - ATF4, IRE1α - XBP1 and ATF6α. First step following the accumulation of unfolded proteins is the activation of BIP and its dissociation from the other membranous stress sensors: PERK, IRE1αand ATF6α 10. Once the dissociation takes place, PERK, IRE1α and ATF6α become activated in a cascade manner. PERK phosphorylates EIF2α and in turn activates ATF4, which up-regulates multiple downstream target molecules, including CHOP. ATF4 activate ER stress response genes that are responsible for the antioxidant reaction and synthesis of the amino acids needed for cell survival 11. ATF4 also activates transcription of CHOP which is required for ER-stress-mediated apoptosis both *in vitro* and *in vivo* 12, 13. In fact, CHOP inhibits BCL2, which is an anti-apoptotic protein. On the other hand, IRE1α activates XBP1 and in turn up-regulates the expression of protein disulfide isomerase (PDI), an enzyme that allows proteins to quickly find the correct arrangement of disulfide bonds in their fully folded state, and therefore acts to catalyze protein folding 14.

The outcome of UPR activation increases protein folding, transport and ER-associated protein degradation (**ERAD**), while attenuating protein synthesis. Such pre-adaptive events, by blocking global protein synthesis, will eventually restore ER homeostasis 15, 16. However, if protein misfolding is not resolved, cells enter apoptosis. Induction of CHOP is an alternative element that becomes activated to switch from pro-adaptive to pro-apoptotic signaling when the damage is severe and prolonged 17, 18. Under conditions of chronic stress, PERK activation leads to apoptosis, as the IRE1α - XBP1 and ATF6α pathways are attenuated 19-21. Therefore, PERK activation promotes both adaptive and apoptotic responses depending on the severity of the stress. The stress response is most likely to be a tissue-specific reaction depending on the threshold of the ER stress tolerance of the tissue 19-21.

Our microarray study revealed a number of functional pathways in the NHOKs that may be altered by EC aerosols. These activated pathways include UPR, protein ubiquitination, oxidative stress response, NF-κB signaling, IL-6 signaling, IL-8 signaling, IL-10 signaling, TGF-β signaling, HGF signaling, cell cycle regulation, EMT regulation, *etc*. Some of these pathways are related to cancer mechanisms or inflammation. Importantly, both microarray and qPCR analyses demonstrated that EC aerosols induce UPR in NHOKs. In fact, both datasets were well correlated with respect to the expression of UPR pathway genes. In response to EC aerosol treatment, CHOP, ATF4, XBP1 (total expression) and IRE1α were found to be significantly over-expressed in NHOKs. Particularly, CHOP was dramatically up-regulated (fold change, 43.1). However, ATF6α gene expression was almost not changed by EC aerosol exposure, as indicated by both microarray and qPCR analyses although its protein expression slightly increased. Whether ATF6 signals are involved in the activation of UPR by EC aerosols remains to be further verified. Western blot analysis also confirmed that GRP78 (BIP), CHOP, ATF4, XBP1s (spliced XBP1) and IRE1α were significantly up-regulated in NHOKs treated with EC aerosols. These results suggest that EC aerosols most likely induce UPR response *via* the mediation of PERK - EIF2α - ATF4 and IRE1α - XBP1 signals. The dramatic over-expression of CHOP also implies that EC aerosols may cause apoptosis of NHOKs *via* the activation of cascade of PERK, EIF2α, ATF4 and CHOP. In fact, recent studies have demonstrated that conventional smoke induces UPR *via* PERK - EIF2α - ATF4 and IRE1α - XBP1 signals 22, 23.

In summary, our study has demonstrated that EC aerosols induce the UPR response in normal human oral epithelial cells mediated by PERK - EIF2α - ATF4 and IRE1α - XBP1 signals. As mentioned earlier, UPR plays important role in restoring homeostasis and assisting protein folding in cells. However, it may also lead to apoptosis under chronic stress and cause cytotoxicity. What chemical components or physiochemical characteristics of EC aerosols induce the UPR signaling remains unknown. It may result from nanoparticles or trace amount of heavy metals present in EC aerosols 8. Some other pathways related to cancer mechanisms or inflammation may also be activated in oral keratinocytes by EC aerosols although this needs to be further verified. EC is generally considered safer than conventional tobacco cigarettes; however, their impact on oral and systemic health remains uncertain. Considering the rapid increase of EC consumption especially among adolescents and young adults, it is important to study potential adverse effect of ECs on the oral health. It should be noted that our *in vitro* experimental setting may not well represent the actual *in vivo* condition for studying the effect of EC aerosols. Animal model studies as well as the investigation of cellular UPR pathways in EC users may confirm our *in vitro* study results and lead to additional findings. In addition, it is highly relevant to compare conventional cigarettes with ECs in terms of their induction of cellular stress responses (*e.g.*, UPR and oxidative stress). These are valid concerns and certainly warrant further studies in the future.

## Figures and Tables

**Table 1:** The primers used for qPCR analysis in this study.

BIP F	TGCTGGCCTAAATGTTATG
BIP R	TGGTGAGAAGAGACACATC
PERK F	CTTATGCCAGACACACAGGACAA
PERK R	TCCATCTGAGTGCTGAATGGAATAC
IRE1a F	GAAGATCCAGTCCTGCAGGTC
IRE1a R	AGAAGAGAGGTTGATGGGCAG
XBP1 F	GTGAGCTGGAACAGCAAGTGGT
XBP1 R	CCAAGCGCTGTCTTAACTCCTG
ATF6 F	GCCGCCGTCCCAGATATTA
ATF6 R	GCAAAGAGAGCAGAATCCCA
CHOP F	TGCTTTCAGGTGTGGTGATGTATG
CHOP R	AATCAGAGCTGGAACCTGAGGA
ATF4 F	AAGCCTAGGTCTCTTAGATG
ATF4 R	TTCCAGGTCATCTATACCCA

**Table 2:** Altered signaling pathways in NHOKs by EC aerosols.

Ingenuity Canonical Pathways	-log(p)	Number of Molecules	Molecules
Unfolded protein response	5.48E00	16	DDIT3,HSPA14,ERN1,INSIG1,XBP1,CEBPB,DNAJB9,HSPA2,HSPA1L,CEBPG,HSPA8,SEL1L,SYVN1,HSPA4,PPP1R15A,ATF4
Glucocorticoid Receptor Signaling	4.25E00	42	ICAM1,TGFBR1,HSPA14,POU2F2,SOS2,GTF2H2,KRAS,HSPA1L,TSC22D3,IL1R2,HSPA4,IKBKB,GTF2B,NFKBIA,NFAT5,HSP90AB1,ANXA1,TGFB2,FKBP5,SERPINE1,ADRB2,CREBZF,CXCL8,CDKN1C,NRAS,TAF5L,CREBBP,SMARCE1,CEBPB,PPP3CC,STAT3,HSPA2,HSPA8,FOS,RRAS2,TAF5,IL1RN,HSP90AA1,NFATC2,IL1B,PLAU,PTGS2
Protein Ubiquitination Pathway	3.62E00	38	B2M,USP24,CRYAB,USP45,HSPA14,CDC20,UBE2N,USP53,ANAPC10,CDC23,DNAJA1,HSPA1L,PAN2,HSPA4,HSP90AB1,UBE2B,USP13,NEDD4L,PSMC2,NEDD4,USP15,USP9X,MDM2,HSPD1,DNAJB9,HSPA2,HSPA12A,SKP2,USP31,HSPA8,USP32,CUL2,HSPA13,ANAPC5,HSP90AA1,DNAJB6,USP9Y,USP25
B Cell Receptor Signaling	3.10E00	27	RAC2,POU2F2,SOS2,INPPL1,KRAS,BCL6,PTK2,IKBKB,NFKBIA,NFAT5,CFL2,ATF4,NRAS,PRKCQ,EGR1,CREBBP,PPP3CC,ATF2,SYNJ2,CALM1 (includes others),RRAS2,DAPP1,BCL10,PAG1,NFATC2,MAP2K3,MAP3K8
Molecular Mechanisms of Cancer	2.98E00	47	FZD10,RAC2,BMP4,TGFBR1,FZD3,ARHGEF7,BMP2,SOS2,CTNNA1,KRAS,FZD1,CDKN2B,CCND1,PTK2,NFKBIA,TGFB2,HIPK2,CDC25A,PMAIP1,NRAS,GNAS,PRKCQ,PAK6,TFDP1,CYCS,ITGA2,PTCH1,CREBBP,SMAD7,CDK6,MDM2,AURKA,FZD8,FOS,CCNE1,CCND2,RRAS2,LEF1,FZD5,MAP2K3,CFLAR,BMP6,NOTCH1,ARHGEF10,FNBP1,GNAL,WNT5A
Cyclins and Cell Cycle Regulation	2.86E00	15	HDAC9,HDAC4,TFDP1,PPP2CA,WEE1,CDK6,CCND1,CDKN2B,SKP2,CCNB1,CCNE1,CCND2,TGFB2,PPP2R1B,CDC25A
Cell Cycle: G1/S Checkpoint Regulation	2.79E00	13	HDAC9,HDAC4,TFDP1,CDK6,MDM2,CCND1,CDKN2B,SKP2,NRG1,CCNE1,CCND2,TGFB2,CDC25A
TGF-β Signaling	2.74E00	16	BMP4,NRAS,TGFBR1,BMP2,CREBBP,SOS2,SMAD7,KRAS,TGIF1,INHBA,FOS,RRAS2,TGFB2,MAP2K3,SERPINE1,TFE3
ILK Signaling	2.73E00	27	SNAI2,FN1,PPP2CA,BMP2,MYH11,RICTOR,CCND1,VEGFA,PTK2,NCK2,TGFB1I1,CFL2,PPAP2B,ATF4,IRS2,ITGB5,NACA,CREBBP,FERMT2,VEGFC,ATF2,DOCK1,FOS,LEF1,PTGS2,PPP2R1B,FNBP1
PPAR Signaling	2.58E00	16	NRAS,PPARD,CREBBP,SOS2,KRAS,IL1R2,IKBKB,FOS,IL18,NFKBIA,RRAS2,HSP90AB1,IL1RN,IL1B,HSP90AA1,PTGS2
NRF2-mediated Oxidative Stress Response	2.55E00	26	NQO2,GCLC,KRAS,DNAJA1,CUL3,HMOX1,SCARB1,ATF4,GCLM,FKBP5,CBR1,NRAS,PRKCQ,NQO1,CREBBP,HERPUD1,JUNB,DNAJB9,BACH1,FOS,RRAS2,STIP1,MAP2K3,SQSTM1,DNAJB6,ABCC4
Ephrin Receptor Signaling	2.42E00	25	RAC2,PTPN13,SOS2,LIMK2,KRAS,VEGFA,PTK2,NCK2,GNB4,CFL2,EFNA5,ATF4,ACTR2,NRAS,GNAS,PAK6,ITGA2,CREBBP,VEGFC,STAT3,EFNA1,ATF2,RRAS2,EPHB3,GNAL
Regulation of IL-2 Expression in Activated and Anergic T Lymphocytes	2.37E00	14	NRAS,TGFBR1,SOS2,KRAS,PPP3CC,IKBKB,CALM1 (includes others),FOS,NFAT5,RRAS2,NFKBIA,BCL10,TGFB2,NFATC2
CDP-diacylglycerol Biosynthesis I	2.12E00	5	TAMM41,GPAM,AGPAT5,ABHD5,AGPAT9
Regulation of the Epithelial-Mesenchymal Transition Pathway	2.10E00	25	FZD10,ID2,SNAI2,TGFBR1,FZD3,SOS2,KRAS,FZD1,FGFR4,TGFB2,HMGA2,TWIST2,NRAS,JAG2,EGR1,STAT3,FZD8,RRAS2,LEF1,FZD5,MAP2K3,FGFRL1,JAG1,NOTCH1,WNT5A
PI3K/AKT Signaling	1.99E00	18	NRAS,PPP2CA,SOS2,ITGA2,MDM2,KRAS,INPPL1,CCND1,EIF4E,SYNJ2,IKBKB,NFKBIA,RRAS2,HSP90AB1,HSP90AA1,MAP3K8,PTGS2,PPP2R1B
Human Embryonic Stem Cell Pluripotency	1.96E00	19	FZD10,TGFBR1,GNAS,BMP4,FZD3,BDNF,BMP2,SMAD7,FZD1,INHBA,S1PR3,FZD8,FGFR4,TGFB2,FZD5,LEF1,FGFRL1,BMP6,WNT5A
NF-κB Signaling	1.93E00	23	AZI2,TGFBR1,BMP4,PRKCQ,NRAS,BMP2,UBE2N,CREBBP,TNFAIP3,KRAS,TANK,IL1R2,IKBKB,IL18,GHR,RRAS2,NFKBIA,IL1RN,BCL10,FGFR4,IL1B,MAP3K8,FGFRL1
PAK Signaling	1.90E00	14	NRAS,PAK6,ARHGEF7,SOS2,ITGA2,KRAS,MYLK,LIMK2,PTK2,NCK2,RRAS2,CFL2,EPHB3,MYL12A
Phosphatidylglycerol Biosynthesis II	1.88E00	5	TAMM41,GPAM,AGPAT5,ABHD5,AGPAT9
IL-6 Signaling	1.85E00	17	CXCL8,SOCS3,NRAS,SOS2,KRAS,STAT3,CEBPB,VEGFA,IL1R2,FOS,IKBKB,IL18,RRAS2,NFKBIA,IL1RN,IL1B,MAP2K3
IL-8 Signaling	1.80E00	24	RAC2,CXCL8,NRAS,ICAM1,GNAS,PRKCQ,HBEGF,VEGFC,LIMK2,KRAS,PLD6,CCND1,PTK2,VEGFA,IKBKB,HMOX1,FOS,GNB4,CCND2,RRAS2,PTGS2,FNBP1,ITGB5,IRAK2
Triacylglycerol Biosynthesis	1.79E00	7	GPAM,AGPAT5,ABHD5,PPP2R2D,PPAP2B,AGPAT9,ELOVL6
IL-10 Signaling	1.65E00	11	IL1R2,HMOX1,IKBKB,SOCS3,FOS,IL18,NFKBIA,IL1RN,IL1B,MAP2K3,STAT3
Sphingosine and Sphingosine-1-phosphate Metabolism	1.64E00	3	ASAH2B,NAAA,SGPP2
HGF Signaling	1.63E00	15	PRKCQ,NRAS,SOS2,ITGA2,KRAS,STAT3,CCND1,ATF2,PTK2,FOS,DOCK1,ELF3,RRAS2,MAP3K8,PTGS2
T Cell Receptor Signaling	1.59E00	14	PRKCQ,NRAS,SOS2,KRAS,PPP3CC,IKBKB,CALM1 (includes others),FOS,NFKBIA,NFAT5,RRAS2,BCL10,PAG1,NFATC2
p53 Signaling	1.53E00	14	HDAC9,PMAIP1,GADD45B,TP63,SNAI2,PIAS1,MDM2,CCND1,CCNG1,CCND2,STAG1,THBS1,HIPK2,DRAM1
PTEN Signaling	1.48E00	16	RAC2,TGFBR1,NRAS,SOS2,ITGA2,INPPL1,KRAS,CCND1,SYNJ2,PTK2,IKBKB,MAGI1,RRAS2,GHR,FGFR4,FGFRL1
CD40 Signaling	1.45E00	10	TANK,IKBKB,FOS,ICAM1,NFKBIA,PTGS1,TNFAIP3,MAP2K3,STAT3,PTGS2

**Table 3:** Differentially expressed genes of the UPR pathway between E-cig aerosol-treated and untreated NHOKs based on DNA microarray analysis.

Gene *#*	Protein name	Protein ID	Average fold change
HSPA5	Immunoglobulin heavy-chain binding protein (a.k.a., glucose-regulated protein 78)	BIP (GRP78)	1.68
ERN1	Inositol-requiring enzyme 1 (a.k.a., endoplasmic reticulum to nucleus signaling 1)	IRE1α	1.97
XBP1	X‑box binding protein 1	XBP1	2.52
EIF2AK3	PRKR-like ER kinase	PERK	1.35
ATF6	Activating transcription factor 6α	ATF6α	1.01
ATF4	Activating transcription factor 4	ATF4	3.65
DDIT3	C/EBP homologous protein (a.k.a., DNA-damage-inducible transcript 3)	CHOP	9.60
